# Antifungal Susceptibility of *Malassezia pachydermatis* Isolates from Companion Animals and Genomic Insights into Resistance Mechanisms

**DOI:** 10.3390/antibiotics14090902

**Published:** 2025-09-05

**Authors:** Marianna Domán, Dávid Első, Krisztina Pintér, Enikő Wehmann, Enikő Fehér, Tibor Magyar

**Affiliations:** 1 HUN-REN Veterinary Medical Research Institute, 1143 Budapest, Hungarymagyar.tibor@vmri.hun-ren.hu (T.M.); 2 National Laboratory of Infectious Animal Diseases, Antimicrobial Resistance, Veterinary Public Health and Food Chain Safety, University of Veterinary Medicine, 1078 Budapest, Hungary; 3 Duo-Bakt Veterinary Microbiology Laboratory, 2112 Veresegyház, Hungary; 4 Department of Microbiology and Infectious Diseases, University of Veterinary Medicine, 1143 Budapest, Hungary

**Keywords:** *Malassezia pachydermatis*, antifungal susceptibility, ergosterol biosynthesis, azoles, *ERG11* mutations

## Abstract

**Background/Objectives**: *Malassezia pachydermatis* is a lipophilic yeast frequently associated with otitis externa and dermatological disorders in companion animals. This study aimed to evaluate the antifungal susceptibility of *M. pachydermatis* isolates from dogs and cats and to investigate the genomic determinants of reduced susceptibility. **Methods**: Susceptibility testing of 87 clinical isolates was performed using a modified CLSI broth microdilution method in Sabouraud dextrose broth supplemented with 1% Tween 80. The whole genome of ten representative isolates was sequenced and the genetic factors that are involved in drug resistance were investigated. **Results**: Ketoconazole, itraconazole, and terbinafine exhibited the highest efficacy, while miconazole and clotrimazole showed reduced activity. Whole genome sequencing revealed single nucleotide polymorphisms (SNPs) in genes that play a key role in the ergosterol biosynthesis pathway, particularly in *ERG11* and *ERG1*. While some specific amino acid substitutions (e.g., K446R in *ERG11*) were found only in isolates with elevated MIC values, no direct correlation with resistance could be unequivocally established. **Conclusions**: Genomic analyses also uncovered chromosomal mutations and the heterozygosity of certain isolates, suggesting that complex, multifactorial mechanisms may drive the development of drug resistance. These findings highlight the importance of standardized susceptibility testing and further genomic investigations to promote effective antifungal therapy in veterinary medicine.

## 1. Introduction

*Malassezia pachydermatis* is a lipophilic, opportunistic yeast that is a component of the normal cutaneous and mucosal microbiota of various domestic animals, especially dogs and cats. Favourable microenvironmental conditions (e.g., a warm and humid environment, oily skin, or the application of oily topical substances) can promote the excessive proliferation of this organism, enabling it to act as an opportunistic secondary pathogen [1]. Clinical manifestations associated with the overgrowth of *M. pachydermatis* include otitis externa and dermatological disorders, such as seborrheic dermatitis. These infections are often recurrent and chronic, posing diagnostic and therapeutic challenges in veterinary practice [1]. In humans, systemic infections have also been reported, particularly in neonates and immunocompromised individuals receiving parenteral nutrition. While lipid infusion and total parenteral nutrition, owing to the lipolytic activity of *Malassezia* spp., are major risk factors for *Malassezia* fungemia, cases also occur without intravenous nutrition. These infections largely affect severely immunocompromised patients with central venous catheters, highlighting this device as a critical portal of fungal invasion [2,3].

The rising clinical relevance of *M. pachydermatis* in companion animals has led to increased attention toward its antifungal susceptibility profiles. Azole antifungals—particularly miconazole, clotrimazole, ketoconazole, and itraconazole—remain the primary therapeutic agents for otitis and dermatitis; although other agents from different chemical classes are also used (terbinafine, thiabendazole) [4]. Nonetheless, some reports of decreased susceptibility and treatment failure have raised concerns regarding the emergence of antifungal resistance, necessitating a comprehensive understanding of the resistance mechanisms and reliable detection methods [4]. Despite the clinical importance of this yeast, a standardized method for the antifungal susceptibility testing of *M. pachydermatis* has not yet been developed. The reference method for testing the susceptibility of yeasts (i.e., *Candida* spp. and *Cryptococcus neoformans*) is inapplicable to *M. pachydermatis* [5,6]. This is primarily attributed to the organism’s fastidious growth requirements (lipid dependency, slow growth, and tendency to form clusters) and the lack of established interpretive breakpoints, both of which hinder accurate resistance assessment and informed therapeutic decision-making. Sabouraud dextrose broth (SDB) supplemented with 1% Tween 80 and Dixon broth are commonly utilized as alternatives to RPMI 1640 in the Clinical and Laboratory Standard Institute (CLSI) broth microdilution (BMD) protocol for *M. pachydermatis* [7]. Although SDB with 1% Tween 80 seems to be an optimal broth medium that supports the adequate growth of *M. pachydermatis*, a liquid medium yielding reliable and reproducible minimum inhibitory concentrations (MICs) in accordance with the CLSI BMD methodology has yet to be definitively established [7,8].

Only a few investigations have been performed to clarify the molecular mechanisms underlying antifungal resistance in *M. pachydermatis* [9,10,11]. These studies have identified several key factors involved in resistance phenotypes. Among them, the overexpression of efflux pump genes, which actively expel antifungal agents from the cell, might play a role in azole resistance. This mechanism reduces intracellular drug concentrations, thereby diminishing antifungal efficacy and contributing to treatment failure. In addition to efflux-mediated resistance, mutations in the *ERG11* gene, which encodes lanosterol-14α-demethylase—an essential enzyme in the ergosterol biosynthesis pathway—have been implicated. Mutations in the *ERG11* gene can lead to structural changes in the target enzyme, reducing the binding affinity of azole antifungals and conferring resistance. Furthermore, chromosomal rearrangements affecting sterol biosynthesis pathways have also been observed [9,10,11]. While research on various fungi has demonstrated that mutations in the *ERG1* gene can result in terbinafine resistance [12,13], no direct evidence currently exists to establish a similar link between *ERG1* mutations and terbinafine resistance in *M. pachydermatis*. Studies have revealed similarities to resistance mechanisms described in other yeast species, although notable species-specific differences persist and warrant further investigation to fully characterize the molecular determinants of resistance and understand their clinical implications that facilitate the development of targeted therapeutic strategies [14,15]. Given the lack of extensive genomic investigations of *M. pachydermatis* in association with reduced antifungal susceptibility, this study aimed to evaluate the antifungal susceptibility of *M. pachydermatis* collected from dogs and cats in Hungary and to investigate the genomic determinants of reduced susceptibility by whole genome sequencing, particularly focusing on the *ERG11* and *ERG1* genes.

## 2. Results

**Figure 1:**
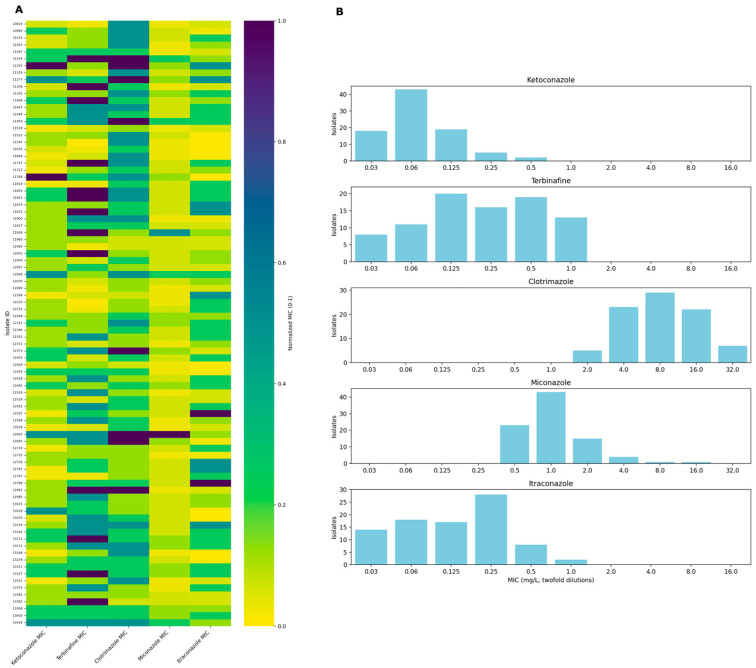
Heatmap of normalized minimum inhibitory concentrations (MICs) and MIC distribution (mg/L) for antifungal agents across *M. pachydermatis* clinical isolates. (**A**): The heatmap illustrates the normalized MICs of five antifungal agents—ketoconazole, terbinafine, clotrimazole, miconazole, and itraconazole—across clinical isolates. MIC values have been normalized on a scale from 0 to 1, with 0 representing the lowest MIC and 1 representing the highest MIC for each antifungal. Each row corresponds to a unique isolate identifier, and each column represents an antifungal agent. The colour gradient from yellow to purple corresponds to increasing normalized MIC values. (**B**): Bar plots show the frequency distribution of MICs for five antifungal agents—ketoconazole, terbinafine, clotrimazole, miconazole, and itraconazole—tested against clinical isolates. MIC values are demonstrated in mg/L as twofold dilutions along the *x*-axis, while the number of isolates corresponding to each MIC value is plotted on the *y*-axis.

Out of 124 isolates, 91 were identified as *M. pachydermatis* and 1 isolate was identified as *Candida parapsilosis* by sequencing. PCR products were not gained from 13 isolates, and 19 isolates did not grow on Sabouraud dextrose agar (SDA). These isolates were excluded from the analysis. Additionally, 4 isolates that were confirmed as *M. pachydermatis* could not be re-grown from frozen culture; therefore, a total of 87 isolates were involved in the study. SDB with 1% Tween 80 proved to be a suitable culture media for CLSI broth microdilution as all isolates were grown sufficiently for visual reading after 72 h incubation. The antifungal susceptibility profiles of the *M. pachydermatis* isolates are summarized in Figure 1 and Table S1. The most effective antifungal drug was ketoconazole with a range of MICs ≤ 0.03–0.5 mg/L (MIC_50_ = 0.06 mg/L, MIC_90_ = 0.125 mg/L). Terbinafine and itraconazole exhibited good antifungal activity against *M. pachydermatis* isolates, with MIC values ranging from 0.03 to 1 mg/L and ≤0.03 to 1 mg/L, respectively. The MIC_50_ values were 0.25 mg/L for terbinafine and 0.125 mg/L for itraconazole. The majority of isolates were inhibited at concentrations of 1 mg/L of terbinafine and 0.5 mg/L of itraconazole. Miconazole demonstrated moderate activity, with an MIC range of 0.5–16 mg/L, an MIC_50_ of 1 mg/L, and an MIC_90_ of 16 mg/L. Clotrimazole was the least effective among the tested antifungal agents, as it required the highest concentrations (2 to >32 mg/L) to inhibit fungal growth. The MIC_50_ and MIC_90_ for clotrimazole were 8 mg/L and 16 mg/L, respectively.

The modal MIC values were 0.06 mg/L, 8 mg/L, 1 mg/L, 0.25 mg/L, and 0.5 mg/L for ketoconazole, clotrimazole, miconazole, itraconazole, and terbinafine, respectively. Considering our dataset, epidemiological cut-off values (ECVs) for ketoconazole (0.25 mg/L), clotrimazole (32 mg/L), miconazole (4 mg/L), itraconazole (1 mg/L), and terbinafine (2 mg/L) were established. Based on the criteria that the ECV should cover at least 95% of the isolates in the wild type (isolate without acquired resistance mechanisms) distribution and isolates included in the “resistant” category are those for which the MIC results are higher than the ECV [4], two isolates, one isolate, and two isolates were resistant to ketoconazole, clotrimazole, and miconazole, respectively. Itraconazole and terbinafine resistance was not detected.

**Figure 2:**
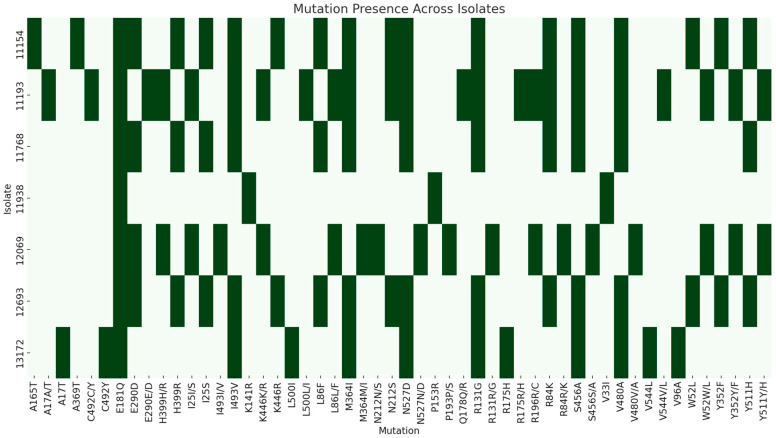
Distribution of amino acid substitutions in the Erg1p and Erg11p among isolates. Isolates with no changes in amino acid sequences are not listed.

The sequencing of *M. pachydermatis* genomes resulted in 5,725,819–13,724,747 reads with 96.8–288.5-fold mean coverage of the six chromosomes. To evaluate the associations between the MIC values and mutations in genes that might be responsible for azole and terbinafine resistance, SNPs in the *ERG11* and *ERG1* genes were analyzed. The *ERG11* sequence length was 1623 bp, while the *ERG1* sequence was 1650 bp. Amino acid substitutions varied among isolates. No changes in amino acid sequence were found in isolates 12747, 11277, and 11450 compared with the reference sequence. However, in other isolates, missense mutations were detected in both genes. Interestingly, hotspot regions were identified where amino acid substitutions frequently occurred. The most prevalent substitution was E181Q (7/10, 70%) in the *ERG11* gene. Isolates with a high clotrimazole MIC value (≥16 mg/L) often carried substitutions I25S, W52L, R84K, L86F, N212S, E290D, Y352F, H399R, and K446R. The amino acid change K446R was specific to isolates with a ≥32 mg/L MIC value (Table 1, Figure 2). In general, isolates with these substitutions showed reduced susceptibility (higher MIC values) to ketoconazole and miconazole, as well, compared with other isolates (*p* ≤ 0.0005). In addition, substitutions I25S, R84K, L86F, E290D, and H399R were always found together in isolates. R84K, N212S, E290D, and H399R substitutions were associated with elevated miconazole MICs (*p* = 0.001–0.009).

**Figure 3:**
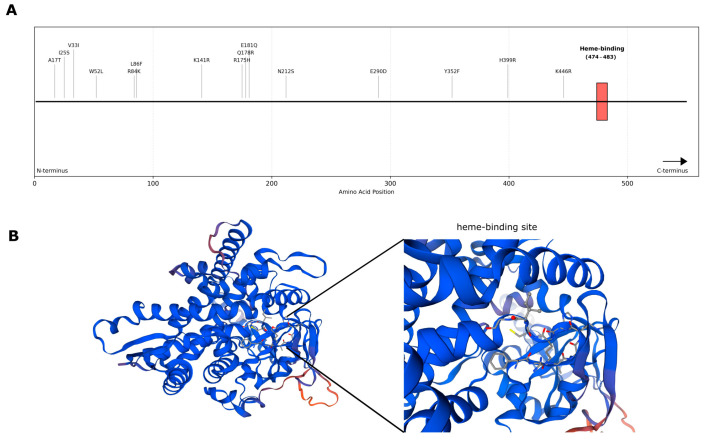
Amino acid substitutions and predicted 3D structure of Erg11p of *M. pachydermatis*. (**A**): Schematic figure of Erg11p (the lanosterol 14α-demethylase enzyme) with the amino acid sequence positions of substitutions identified in this study. Heme-binding site is marked with a red rectangle. (**B**): Predicted tertiary structure of the Erg11p protein of isolate 12693 generated by homology modelling (SWISS-MODEL), highlighting key structural features, including the heme-binding domain (FGAGRHRCIG motif) [16].

We investigated whether these substitutions were located in the azole-binding region; thus, the specific sequence of the putative heme-binding site of *M. pachydermatis* (^474^FGAGRHRCIG^483^) was identified based on sequence data gained from other *Malassezia* species [16]. None of the residue changes in Erg11p were located in or near the heme-binding site and no significant correlation was found between high azole MIC values and amino acid substitutions (Figure 3). Interestingly, in the case of isolates 11193 and 12069, we observed that two different allele types were detected. In the Erg1p, more than half of the isolates possessed R131G, M364I, S456A, V480A, I493V, and N527D substitutions. There was no evidence that any of the substitutions resulted in higher MIC values; however, the heterozygosity of isolates 11193 and 12069 was observed (Table 2, Figure 2). Similar genomic patterns were noticed regarding hotspot regions and different allele types in the other genes that play a role in the ergosterol biosynthesis pathway (Table S2). The annotation of *ERG2* in the reference sequence was inappropriate; therefore, we excluded this gene from the analysis. Of note, frameshift mutations (homozygous mutations) were detected in the *ERG7* gene of isolates 11154, 11768, 12693, and 13171. Due to frameshift, the *ERG7* gene of isolate 13172 was 1806 bp, while the *ERG7* gene of isolates 12693, 11154, and 11768 was 1848 bp long (94 bp and 52 bp shorter than those of other isolates, respectively). Nevertheless, these mutations were located in the C-terminal of the protein, yielding a truncated amino acid sequence; hence, the functional loss of the protein is unlikely. In *ERG24*, two indels (from 911 to 936 bp and from 1166 to 1242 bp) were detected in isolates 11154, 11193, 11277, 11450, 12069, and 13172. The first indel (homozygous) resulted in a stop codon at nucleotide position 930, thus two coding sequences were found instead of one. Overall, the second indel (heterozygous in 11193 and 12069) generated protein truncation in isolates; however, substantial truncation was observed only in isolates 11277, 11450, and 12069. These changes in *ERG24* (two coding sequences and truncations) might affect protein function.

## 3. Discussion

The assessment of the antifungal resistance of *M. pachydermatis* poses considerable difficulties as there are no standardized methodologies for susceptibility testing, and no breakpoints for classifying isolates as susceptible or resistant [8]. Establishing a set of standardized criteria for in vitro testing is crucial as different testing variables have a significant impact on in vitro evaluation. We followed the general principles for the susceptibility testing of yeasts developed by the CLSI with some modification to meet the special growth requirements of *M. pachydermatis*. We used SDB with 1% Tween 80 as it was proposed as the most suitable medium for the broth microdilution protocol [7]. We also initiated some adaptations for the efficient visual reading of results (the dilution of inoculum, incubation time). Our results demonstrated that *M. pachydermatis* isolates were more susceptible to ketoconazole, itraconazole, and terbinafine, and less susceptible to miconazole and clotrimazole. These observations are in accordance with previous findings [7,17,18,19,20]; however, we determined a slightly higher MIC_90_ values for ketoconazole (0.125 mg/L vs. 0.03 or 0.06 mg/L), itraconazole (0.5 mg/L vs. <0.008 or 0.016 mg/L), and terbinafine (1 mg/L vs. 0.25 mg/L). Regardless of the variability of test conditions in several studies, the most effective drugs were ketoconazole (MIC_90_ ≤ 1 mg/L), itraconazole, and posaconazole (MIC_90_ for most studies ≤ 0.5 mg/L) [4]. Based on data from the literature, fluconazole had higher mean MIC values (range 4–>64 mg/L) than other azole derivatives and it proved to be the less effective agent against *M. pachydermatis* [18,20].

Despite the absence of established breakpoints, significantly elevated MIC values may indicate resistance since treatment failure has been reported after azole administration [21,22]. Angileri et al. observed that the prolonged systemic and topical azole treatment of recurrent *Malassezia* dermatitis and otitis in a 5-year-old toy Poodle resulted in multi-azole resistance [21]. Both the BMD and E-test method showed elevated ketoconazole and itraconazole MIC values for the six isolates obtained from the skin and right ear of the toy Poodle compared with the control ones. Notably, one isolate exhibited marked resistance to both miconazole and itraconazole. Intriguingly, azole antifungal agents that had not been previously administered to the affected dog—namely posaconazole, fluconazole, and ketoconazole—also demonstrated reduced in vitro activity, indicating a potential cross-resistance of *M. pachydermatis* to multiple azole compounds [21]. In another study, strains of *M. pachydermatis* with low susceptibility to several azoles were also found, regardless of the health status of the dogs investigated (diseased or healthy) [23].

In contrast to *Candida* species and human-pathogenic *Malassezia* species, the mechanisms underlying antifungal resistance in *M. pachydermatis* remain relatively poorly characterized. Kano et al. detected missense mutations in the *ERG11* gene encoding lanosterol 14α-demethylase, which is the target enzyme of azole antifungal drugs. Nucleotide substitutions A412G (amino acid substitutions M138V) and C905T (V302A) resulted in itraconazole resistance (MIC = >32 mg/L), while G1382A (G461N) was associated with ravuconazole resistance (MIC = >32 mg/L) [22,24]. This latter finding is particularly worrisome given that ravuconazole is authorized solely for use in human medicine and is not currently used for the treatment of *Malassezia* infections in animals. Therefore, understanding the molecular basis of antifungal drug resistance is of utmost importance. Díaz et al. revealed the high diversity of *ERG11* gene sequences of 31 *M. pachydermatis* strains with amino acid substitutions considered as neutral (A17T, I25V, I25S, V33I, R84K, L86F, D166E, R175H, Q178R, E181Q, N212S, E290D, T354I, H399R, D405N) or deleterious (W52L, F143S, R202H, S226L, A302T, A306S, Y352F, G459D, G461D) [10]. We observed similar variability of the *ERG11* gene of the sequenced isolates; however, these amino acid substitutions (such as W52L and Y352F) were not associated with reduced azole susceptibility. Nonetheless, in the Spanish study, the E-test was used for susceptibility testing and only in the case of fluconazole were associations between mutations and resistance found, indicating that the mechanisms of resistance in *M. pachydermatis* are complex and require widespread genomic studies. Despite our finding that K446R homozygous substitution was detected exclusively in isolates 11154 and 12693 with a ≥32 mg/L clotrimazole MIC value, the role of this substitution in resistance remains questionable, because other pathways that might play a role cannot be ruled out.

Kim et al. investigated the mechanism underlying ketoconazole resistance (MIC = 8 mg/L) in a clinical isolate of *M. pachydermatis*. Based on whole genome sequencing results, a ~84 kb region in chromosome 4 was tandemly quadruplicated, which included both the *ERG4* and *ERG11* genes. This genomic rearrangement yielded the overexpression of *ERG* genes, which explain the adaptation of *M. pachydermatis* to the selective pressure exerted by ketoconazole [11]. In isolates 11193 and 12069 we detected the heterozygosity of *ERG* genes indicating that two allele types of these genes are present in the investigated *M. pachydermatis* genomes. To the best of our knowledge, there is no available data on aneuploidies in *M. pachydermatis*. However, the genomic evidence of cell–cell fusion leading to a diploid or aneuploid state was found in *Malassezia furfur* and other yeasts [25,26,27,28], raising the possibility that similar evolutionary events could occur *in M. pachydermatis* as well. Further research that employs methods such as flow cytometry or allele frequency analysis could confirm the ploidy status of the isolates and give evidence of genome duplication events or aneuploidies of *M. pachydermatis*.

The upregulation of genes encoding drug transporter classes (efflux pumps) has been associated with the development of resistance in *Candida* species [29]. Increased drug efflux in a ketoconazole-resistant isolate of *Malassezia restricta* has also been reported [15]. Two major classes of efflux pumps have been characterized in yeasts: ATP-binding cassette (ABC) transporters, which utilize ATP hydrolysis as an energy source, and pumps of the major facilitator superfamily, which rely on membrane potential to mediate efflux. Iatta et al. suggested that the resistance mechanisms employed by *M. pachydermatis* against azoles may involve efflux pumps. This hypothesis was supported by in vitro experiments demonstrating enhanced antifungal activity when fluconazole was combined with an efflux pump inhibitor, haloperidol [9]. We identified ABC transporters in the 10 *M. pachydermatis* genomes; however, further investigations are needed to determine their role in antifungal drug resistance.

Terbinafine is classified within the allylamine group of antifungal agents and exerts its mechanism of action by inhibiting an early stage of ergosterol biosynthesis. The squalene epoxidase targeted by terbinafine is encoded by the *ERG1* gene. Although terbinafine resistance related to *ERG1* gene mutations was detected mainly in dermatophytes [13,30], certain yeast species have also demonstrated the capacity to develop resistance mechanisms [12]. On the other hand, no cases of terbinafine resistance have been reported in *M. pachydermatis* so far. This observation was further supported by our results, as neither high MIC values nor *ERG1* substitutions corresponding with resistance were detected. It is noteworthy that several polymorphisms were observed in the *ERG1* gene, which raises the possibility that certain mutations within the *ERG1* may lead to the development of resistance in *M. pachydermatis* strains as well. The genome plasticity noticed in our study through other genes involved in ergosterol biosynthesis indicates that alterations in enzyme cascade could occur; therefore, alternative pathways could develop as seen in *Candida* species [31,32].

Overall, despite the fact that we detected a few alterations in ergosterol biosynthesis genes, the substitutions identified in this study were not directly associated with in vitro resistance. Further functional studies (e.g., site-directed mutagenesis) are necessary to confirm their role in the reduced antifungal susceptibility of *M. pachydermatis*.

## 4. Materials and Methods

### 4.1. Isolate Collection and Species-Level Identification

A total of 124 fungal isolates were collected from September to November 2020 from samples sent for routine diagnostics. Isolates originated from dogs (*n* = 116) and cats (*n* = 8) were obtained from Duo-Bakt Veterinary Microbiology Laboratory. Ear (*n* = 122), tympanum (*n* = 1), and skin (*n* = 1) samples were cultured on Sabouraud dextrose agar (SDA) supplemented with sterile olive oil, chloramphenicol, and gentamicin. Agar plates were incubated at 35 °C for 72 h, then isolates were identified based on morphological characteristics and by sequencing the internal transcribed spacer (ITS) region of nuclear ribosomal DNA [33,34,35]. Briefly, the genomic DNA template used for PCR was extracted from fungal colonies by heat inactivation. The PCR was carried out in a final volume of 25 µL with the reaction containing 3 µL DNA, 2.5 µL 10× Dream-Taq buffer, 0.5 µL dNTP (10 mM), 1 µL forward (5′-TCCGTAGGTGAACCTGCGG-3′) and reverse primers (5′-TCCTCCGCTTATTGATATGC-3′) (10 µM each), 0.15 µL DreamTaq DNA polymerase (5 U/µL; Thermo Fisher Scientific, Waltham, MA, USA), and 16.85 µL nuclease-free water. The thermal cycling protocol included an initial denaturation step at 95 °C for 3 min, followed by 40 cycles of 95 °C for 30 s, annealing at 50 °C for 30 s, extension at 72 °C for 1 min, and a final extension step at 72 °C for 10 min. Amplicons were checked on a 1% agarose gel, then the purified products were sequenced on both strands with a BigDye Terminator v3.1. cycle sequencing kit (Thermo Fisher Scientific, Waltham, MA, USA) on an ABI Prism 3130 Genetic Analyzer (Applied Biosystems, Thermo Fisher Scientific, Waltham, MA, USA). Sequences were edited and assembled using Mega software (version 11) [36]. Species-level identification was performed by the BLAST sequence analysis tool (https://blast.ncbi.nlm.nih.gov/Blast.cgi, accessed on 9 June 2021). Pure cultures of each isolate grown on SDA were stored at −70 °C until further use.

### 4.2. Antifungal Susceptibility Testing

The antifungal susceptibility testing of *M. pachydermatis* isolates was performed using the CLSI broth microdilution protocol [5] with the following modifications. The initial concentration of fungal cell suspension in sterile saline was adjusted to an optical density of 2.4 McFarland [7,17]. The working suspension was made by a 1:100 dilution followed by a 1:5 dilution of the stock suspension in sterile saline. The activity of ketoconazole (0.03–16 mg/L), clotrimazole (0.06–32 mg/L), miconazole (0.06–32 mg/L), itraconazole (0.03–16 mg/L), and terbinafine (0.03–16 mg/L) against *M. pachydermatis* isolates was determined. Twofold dilutions of the drug solutions in SDB with 1% Tween 80 (100 µL) were dispensed into 96-well plates. We used SDB with 1% Tween 80 since, in previous studies, MIC values were more indicative of the susceptible or resistant category in this medium than others (such as Dixon broth or Christensen’s urea broth) [7]. Next, 100 µL of the final inoculum dilution was added to each well except for the negative control (medium only). Quality control strains (*Candida parapsilosis* ATCC 22019 and *Pichia kudriavzevii* ATCC 6258) were involved in each experiment to evaluate the accuracy of the antifungal drug dilutions and to ensure the reproducibility of the results. The visual reading of plates was carried out after 72 h of incubation at 33 °C. The MIC endpoint was defined as the lowest concentration that resulted in a predominant decrease in turbidity (>90%) compared with that of the drug-free control. The MIC determination of antifungal drugs was carried out in triplicate. The concentration of antimycotics that inhibited the growth of 50% and 90% of tested isolates was considered as MIC_50_ and MIC_90_ values, respectively.

### 4.3. Genome Sequencing and Bioinformatics Analysis

Ten representative *M. pachydermatis* isolates were chosen for whole genome sequencing. Isolates with the lowest MIC (12747) and highest MIC (12693) of most antifungal drugs tested were involved in genome sequencing. Other isolates were selected according to the origin of the samples (different breed), variable MIC values, and the appropriate growth in liquid media for genomic DNA extraction. Isolates were grown on SDA at 33 °C for five days. Colonies were inoculated into SDB supplemented with chloramphenicol and 1% Tween 80 and incubated at 33 °C for 24 h. Genomic DNA was extracted from cells with a Quick-DNA Fungal/Bacterial Miniprep Kit (Zymo Research, Irvine, CA, USA) in accordance with the manufacturer’s instructions. The whole genome sequencing of the isolates was conducted by SeqOmics Biotechnology Ltd. (Mórahalom, Hungary) using the Illumina MiSeq sequencing platform (San Diego, CA, USA). Paired-end reads were quality controlled with FastQC and trimmed using the BBDuk algorithm implemented in Geneious Prime (version 2023.2.1). Genome assembly was performed by mapping the trimmed reads to the *M. pachydermatis* reference genome (CBS 1879) using Geneious Prime. Genes encoding different enzymes that are involved in the ergosterol biosynthesis pathway were chosen for single nucleotide polymorphism (SNP) analysis. Multiple sequence alignments of each gene (*ERG1*, *ERG2*, *ERG3*, *ERG4*, *ERG5*, *ERG6*, *ERG7*, *ERG9*, *ERG11*, *ERG24*, *ERG25*, *ERG26*, *ERG27*) were performed using MUSCLE and visualized using Geneious Prime. SNPs that yielded amino acid substitutions compared with the reference were investigated to evaluate the sequence diversity of genes among different *M. pachydermatis* isolates and to determine whether these changes correlate with increased MIC values. We defined wild type as “–/blank” at the locus and compared MIC distributions for mutant versus wild-type isolates with Mann–Whitney U tests. We first tested the effect of any substitution across drugs, then evaluated individual substitutions (restricted to those present in ≥5 isolates) using one-sided tests (alternative = greater) to detect higher MICs. Statistical analysis was performed using Julius AI [37]. Significance was defined as a *p* value of <0.05.

## 5. Conclusions

In general, Hungarian isolates were susceptible to ketoconazole, itraconazole, and terbinafine; however, miconazole and clotrimazole are not recommended as therapeutic agents. It would have been possible to examine potential temporal changes in the susceptibility of the strains if we had worked with a larger number of samples collected over several years. Nevertheless, significant increases in MIC values were not detected, indicating that strains with resistant phenotypes are not prevalent. Although our study has some limitations (a short sampling period, a small number of sequenced isolates), we identified the diverse genomic characteristics of *M. pachydermatis*, which contribute to a deeper understanding of the molecular mechanisms underlying the survival of this yeast under various selective pressures. The genome plasticity of *M. pachydermatis* facilitates the development of resistance, and resistance mechanisms may differ between strains; therefore, extensive genomic and transcriptomic analyses, as well as functional studies are essential to elucidate the species-specific mechanisms of resistance and for guiding evidence-based antifungal use in veterinary practice.

## Figures and Tables

**Table 1:** Antifungal susceptibility of ten *M. pachydermatis* isolates chosen for the purpose of whole genome sequencing and the amino acid substitutions found in the *ERG11* sequence.

Isolate	Position	Substitution	MIC KTZ (mg/L)	MIC TER (mg/L)	MIC CLT (mg/L)	MIC MCZ (mg/L)	MIC ITR (mg/L)
11938	33	V → I	0.06	1	4	8	0.125
	141	K → R					
	181	E → Q					
12069	25	I → I/S	0.25	0.125	16	4	0.25
	52	W → W/L					
	84	R → R/K					
	86	L → L/F					
	181	E → Q					
	212	N → N/S					
	290	E → D					
	352	Y → Y/F					
	399	H → H/R					
	446	K → K/R					
12693	25	I → S	0.25	0.5	>32	16	0.125
	52	W → L					
	84	R → K					
	86	L → F					
	181	E → Q					
	212	N → S					
	290	E → D					
	352	Y → F					
	399	H → R					
	446	K → R					
13172	17	A → T	0.06	0.5	16	2	0.25
	175	R → H					
	181	E → Q					
11154	25	I → S	0.125	1	32	4	0.125
	52	W → L					
	84	R → K					
	86	L → F					
	181	E → Q					
	212	N → S					
	290	E → D					
	352	Y → F					
	399	H → R					
	446	K → R					
11193	17	A → A/T	0.5	0.125	32	2	0.5
	25	I → I/S					
	52	W → W/L					
	84	R → K					
	86	L → L/F					
	175	R → R/H					
	178	Q → Q/R					
	181	E → Q					
	212	N → S					
	290	E → E/D					
	352	Y → Y/F					
	399	H → H/R					
	446	K → K/R					
11768	25	I → S	0.5	0.25	16	2	≤0.03
	84	R → K					
	86	L → F					
	181	E → Q					
	290	E → D					
	399	H → R					
11277	-	-	0.25	0.25	32	2	0.5
11450	-	-	0.125	0.5	32	4	0.25
12747	-	-	≤0.03	0.03	4	1	0.25

CLT: clotrimazole, ITR: itraconazole, KTZ: ketoconazole, MCZ: miconazole, TER: terbinafine.

**Table 2:** Antifungal susceptibility of ten *M. pachydermatis* isolates chosen for the purpose of whole genome sequencing and the amino acid substitutions found in the *ERG1* sequence.

Isolate	Position	Substitution	MIC KTZ (mg/L)	MIC TER (mg/L)	MIC CLT (mg/L)	MIC MCZ (mg/L)	MIC ITR (mg/L)
11938	153	P → R	0.06	1	4	8	0.125
12069	131	R → R/G	0.25	0.125	16	4	0.25
	193	P → P/S					
	196	R → R/C					
	364	M → M/I					
	456	S → S/A					
	480	V → V/A					
	493	I → I/V					
	511	Y → Y/H					
	527	N → N/D					
12693	131	R → G	0.25	0.5	>32	16	0.125
	364	M → I					
	456	S → A					
	480	V → A					
	493	I → V					
	511	Y → H					
	527	N → D					
13172	96	V → A	0.06	0.5	16	2	0.25
	131	R → G					
	364	M → I					
	456	S → A					
	480	V → A					
	492	C → Y					
	493	I → V					
	500	L → I					
	527	N → D					
	544	V → L					
11154	131	R → G	0.125	1	32	4	0.125
	165	A → T					
	364	M → I					
	369	A → T					
	456	S → A					
	480	V → A					
	493	I → V					
	511	Y → H					
	527	N → D					
11193	131	R → G	0.5	0.125	32	2	0.5
	196	R → R/C					
	364	M → I					
	456	S → A					
	480	V → A					
	492	C → C/Y					
	493	I → V					
	500	L → L/I					
	511	Y → Y/H					
	527	N → D					
	544	V → V/L					
11768	131	R → G	0.5	0.25	16	2	≤0.03
	364	M → I					
	456	S → A					
	480	V → A					
	493	I → V					
	511	Y → H					
	527	N → D					
11277	-	-	0.25	0.25	32	2	0.5
11450	-	-	0.125	0.5	32	4	0.25
12747	-	-	≤0.03	0.03	4	1	0.25

CLT: clotrimazole, ITR: itraconazole, KTZ: ketoconazole, MCZ: miconazole, TER: terbinafine.

## Data Availability

Nucleotide sequences of the ITS region of isolates involved in SNP analysis were deposited in GenBank under the following accession numbers: PX113076-PX113085. Raw reads of sequenced *M. pachydermatis* isolates have been deposited to NCBI Sequence Read Archive under BioProject ID PRJNA1291951.

## Supplementary Materials

The following supporting information can be downloaded at: https://www.mdpi.com/article/10.3390/antibiotics14090902/s1, Table S1: In vitro activity of ketoconazole, clotrimazole, miconazole, itraconazole, and terbinafine against 87 M. pachydermatis isolates from dogs and cats with otitis externa and dermatitis; Table S2: Occurrence of amino acid substitutions in proteins responsible for ergosterol biosynthesis and the MIC values of ketoconazole, clotrimazole, miconazole, itraconazole, and terbinafine determined against M. pachydermatis isolates.

## References

[1] Velegraki A., Cafarchia C., Gaitanis G., Iatta R., Boekhout T. (2015). *Malassezia* Infections in Humans and Animals: Pathophysiology, Detection, and Treatment. PLoS Pathog..

[2] Chow N.A., Chinn R., Pong A., Schultz K., Kim J., Gade L., Jackson B.R., Beer K.D., Litvintseva A.P. (2020). Use of Whole-Genome Sequencing to Detect an Outbreak of *Malassezia pachydermatis* Infection and Colonization in a Neonatal Intensive Care Unit—California, 2015–2016. Infect. Control Hosp. Epidemiol..

[3] Rhimi W., Theelen B., Boekhout T., Otranto D., Cafarchia C. (2020). *Malassezia* spp. Yeasts of Emerging Concern in Fungemia. Front. Cell. Infect. Microbiol..

[4] Peano A., Johnson E., Chiavassa E., Tizzani P., Guillot J., Pasquetti M. (2020). Antifungal Resistance Regarding *Malassezia pachydermatis*: Where Are We Now?. J. Fungi.

[5] (2008). Reference Method for Broth Dilution Antifungal Susceptibility Testing of Yeasts.

[6] (2012). Reference Method for Broth Dilution Antifungal Susceptibility Testing of Yeasts.

[7] Cafarchia C., Figueredo L.A., Favuzzi V., Surico M.R., Colao V., Iatta R., Montagna M.T., Otranto D. (2012). Assessment of the Antifungal Susceptibility of *Malassezia pachydermatis* in Various Media Using a CLSI Protocol. Vet. Microbiol..

[8] Peano A., Pasquetti M., Tizzani P., Chiavassa E., Guillot J., Johnson E. (2017). Methodological Issues in Antifungal Susceptibility Testing of *Malassezia pachydermatis*. J. Fungi.

[9] Iatta R., Puttilli M.R., Immediato D., Otranto D., Cafarchia C. (2017). The Role of Drug Efflux Pumps in *Malassezia pachydermatis* and *Malassezia furfur* Defence Against Azoles. Mycoses.

[10] Díaz L., Castellá G., Bragulat M.R., Cabañes F.J. (2023). ERG11 Gene Variability and Azole Susceptibility in *Malassezia pachydermatis*. Mycopathologia.

[11] Kim M., Cho Y.-J., Park M., Choi Y., Hwang S.Y., Jung W.H. (2018). Genomic Tandem Quadruplication Is Associated with Ketoconazole Resistance in *Malassezia pachydermatis*. J. Microbiol. Biotechnol..

[12] Klobučníková V., Kohút P., Leber R., Fuchsbichler S., Schweighofer N., Turnowsky F., Hapala I. (2003). Terbinafine Resistance in a Pleiotropic Yeast Mutant Is Caused by a Single Point Mutation in the *ERG1* Gene. Biochem. Biophys. Res. Commun..

[13] Burmester A., Hipler U., Uhrlaß S., Nenoff P., Singal A., Verma S.B., Elsner P., Wiegand C. (2020). Indian *Trichophyton mentagrophytes* Squalene Epoxidase *Erg1* Double Mutants Show High Proportion of Combined Fluconazole and Terbinafine Resistance. Mycoses.

[14] Zhang J., Li L., Lv Q., Yan L., Wang Y., Jiang Y. (2019). The Fungal CYP51s: Their Functions, Structures, Related Drug Resistance, and Inhibitors. Front. Microbiol..

[15] Park M., Cho Y.-J., Lee Y.W., Jung W.H. (2020). Genomic Multiplication and Drug Efflux Influence Ketoconazole Resistance in *Malassezia restricta*. Front. Cell. Infect. Microbiol..

[16] Kim D., Lim Y.-R., Ohk S.O., Kim B.J., Chun Y.-J. (2011). Functional Expression and Characterization of CYP51 from Dandruff-Causing *Malassezia globosa*: *Malassezia globosa* CYP51. FEMS Yeast Res..

[17] Cafarchia C., Figueredo L.A., Iatta R., Montagna M.T., Otranto D. (2012). In Vitro Antifungal Susceptibility of *Malassezia pachydermatis* from Dogs with and Without Skin Lesions. Vet. Microbiol..

[18] Cafarchia C., Iatta R., Immediato D., Puttilli M.R., Otranto D. (2015). Azole Susceptibility of *Malassezia pachydermatis* and *Malassezia furfur* and Tentative Epidemiological Cut-Off Values. Med. Mycol..

[19] Peano A., Beccati M., Chiavassa E., Pasquetti M. (2012). Evaluation of the Antifungal Susceptibility of *Malassezia pachydermatis* to Clotrimazole, Miconazole and Thiabendazole Using a Modified CLSI M27-A3 Microdilution Method. Vet. Dermatol..

[20] Jerzsele Á., Balázs B., Kálmánfi E., Lajos Z., Gálfi P., Gyetvai B. (2013). Kutyából és macskából izolált *Malassezia pachydermatis* törzsek in vitro érzékenységi vizsgálata. Magy. Állatorvosok Lapja.

[21] Angileri M., Pasquetti M., De Lucia M., Peano A. (2019). Azole Resistance of *Malassezia pachydermatis* Causing Treatment Failure in a Dog. Med. Mycol. Case Rep..

[22] Kano R., Yokoi S., Kariya N., Oshimo K., Kamata H. (2019). Multi-Azole-Resistant Strain of *Malassezia pachydermatis* Isolated from a Canine *Malassezia* Dermatitis. Med. Mycol..

[23] Murayama N., Kano R. (2023). Azole and Terbinafine Susceptibility Testing of *Malassezia pachydermatis* in Japan. J. Vet. Med. Sci..

[24] Kano R., Aramaki C., Murayama N., Mori Y., Yamagishi K., Yokoi S., Kamata H. (2019). High Multi-Azole-Resistant *Malassezia pachydermatis* Clinical Isolates from Canine *Malassezia* Dermatitis. Med. Mycol..

[25] Domán M., Kaszab E., Laczkó L., Bali K., Makrai L., Kovács R., Majoros L., Bányai K. (2024). Genomic Epidemiology of Antifungal Resistance in Human and Avian Isolates of *Candida albicans*: A Pilot Study from the One Health Perspective. Front. Vet. Sci..

[26] Forche A., Alby K., Schaefer D., Johnson A.D., Berman J., Bennett R.J. (2008). The Parasexual Cycle in *Candida albicans* Provides an Alternative Pathway to Meiosis for the Formation of Recombinant Strains. PLoS Biol..

[27] Pryszcz L.P., Németh T., Saus E., Ksiezopolska E., Hegedűsová E., Nosek J., Wolfe K.H., Gacser A., Gabaldón T. (2015). The Genomic Aftermath of Hybridization in the Opportunistic Pathogen *Candida metapsilosis*. PLoS Genet..

[28] Theelen B., Mixão V., Ianiri G., Goh J.P.Z., Dijksterhuis J., Heitman J., Dawson T.L., Gabaldón T., Boekhout T. (2022). Multiple Hybridization Events Punctuate the Evolutionary Trajectory of *Malassezia furfur*. mBio.

[29] Cannon R.D., Lamping E., Holmes A.R., Niimi K., Baret P.V., Keniya M.V., Tanabe K., Niimi M., Goffeau A., Monk B.C. (2009). Efflux-Mediated Antifungal Drug Resistance. Clin. Microbiol. Rev..

[30] Kong X., Tang C., Singh A., Ahmed S.A., Al-Hatmi A.M.S., Chowdhary A., Nenoff P., Gräser Y., Hainsworth S., Zhan P. (2021). Antifungal Susceptibility and Mutations in the Squalene Epoxidase Gene in Dermatophytes of the *Trichophyton mentagrophytes* Species Complex. Antimicrob. Agents Chemother..

[31] Martel C.M., Parker J.E., Bader O., Weig M., Gross U., Warrilow A.G.S., Rolley N., Kelly D.E., Kelly S.L. (2010). Identification and Characterization of Four Azole-Resistant *Erg3* Mutants of *Candida albicans*. Antimicrob. Agents Chemother..

[32] Branco J., Ola M., Silva R.M., Fonseca E., Gomes N.C., Martins-Cruz C., Silva A.P., Silva-Dias A., Pina-Vaz C., Erraught C. (2017). Impact of *ERG3* Mutations and Expression of Ergosterol Genes Controlled by *UPC2* and *NDT80* in *Candida parapsilosis* Azole Resistance. Clin. Microbiol. Infect..

[33] Schoch C.L., Seifert K.A., Huhndorf S., Robert V., Spouge J.L., Levesque C.A., Chen W., Bolchacova E., Fungal Barcoding Consortium, Fungal Barcoding Consortium Author List (2012). Nuclear Ribosomal Internal Transcribed Spacer (ITS) Region as a Universal DNA Barcode Marker for *Fungi*. Proc. Natl. Acad. Sci. USA.

[34] Domán M., Makrai L., Lengyel G., Kovács R., Majoros L., Bányai K. (2021). Molecular Diversity and Genetic Relatedness of *Candida albicans* Isolates from Birds in Hungary. Mycopathologia.

[35] Cafarchia C., Gasser R.B., Figueredo L.A., Latrofa M.S., Otranto D. (2011). Advances in the Identification of *Malassezia*. Mol. Cell. Probes.

[36] Tamura K., Stecher G., Kumar S. (2021). MEGA11: Molecular Evolutionary Genetics Analysis Version 11. Mol. Biol. Evol..

[37] Caesar Labs, Inc. 2025 Julius (June 19 Version) [Large Language Model]. https://julius.ai/.

