# Supplementary material for: Antifungal Susceptibility of Malassezia pachydermatis Isolates from Companion Animals and Genomic Insights into Resistance Mechanisms
Source: Antibiotics (Basel). 2025 Sep 5;14(9):902. doi: 10.3390/antibiotics14090902 (PMC12466836; doi:10.3390/antibiotics14090902)
Supplement: Supplementary file 1 [file antibiotics-14-00902-s001.zip › Table S1.pdf]

Supplementary Table S1. *In vitro* activity of ketoconazole. clotrimazole. miconazole. itraconazole and terbinafine against 87 *M. pachydermatis* isolates from dogs and cats with otitis externa and dermatitis.

Isolate number	Host	Breed	Sex	Age	Sampling site	Location	KTZ MIC (mg/L)	TER MIC (mg/L)	CLT MIC (mg/L)	MCZ MIC (mg/L)	ITR MIC (mg/L)
**10824**	dog	NA	NA	NA	ear	Budapest	≤0.06	≤0.06	16	0.5	0.06
**10990**	dog	Cocker Spaniel	NA	9 years	ear	Budapest	0.125	0.125	16	1	≤0.06
**11014**	dog	NA	NA	NA	ear	Ecser	≤0.06	0.125	16	1	0.25
**11063**	dog	Spaniel	male	7 years	ear	Budapest	≤0.06	0.125	16	0.5	0.125
**11087**	dog	English Cocker Spaniel	male	3 years	ear	Győr	0.125	0.25	8	0.5	0.06
**11306**	dog	Beagle	female	1.5 years	ear	Budapest	0.03	1	8	0.5	0.06
**11277**	dog	Komondor	male	3 years	ear	Veresegyház	0.25	0.25	32	2	0.5
**11335**	dog	NA	NA	NA	ear	Sopron	0.06	0.125	16	2	0.25
**11408**	cat	NA	male	6 years	ear	Siófok	0.125	1	8	1	0.125
**11422**	dog	Jack Russel Terrier	female	8 years	ear	Szeged	0.06	0.5	16	1	0.25
**11448**	dog	Bichon Havanese	female	15 years	ear	Budapest	0.06	0.5	8	1	0.25
**11450**	dog	NA	NA	NA	ear	Budapest	0.125	0.5	32	4	0.25
**11154**	dog	American Staffordshire Terrier	female	6 years	ear	Kóny	0.125	1	32	4	0.125
**11193**	dog	Cross-breed	female	4 years	ear	Budapest	0.5	0.125	32	2	0.5
**11518**	dog	NA	NA	NA	ear	Budapest	≤0.03	0.06	4	0.5	0.06
**11219**	dog	NA	NA	NA	ear	Érd	0.06	0.06	16	1	0.125
**11552**	dog	Cross-breed	male	NA	ear	Aszód	0.06	0.125	16	1	≤0.03
**11597**	dog	Yorkshire Terrier	female	6 years	ear	Budapest	0.06	≤0.03	16	0.5	≤0.03
**11632**	dog	NA	NA	NA	ear	Ecser	0.03	0.03	8	0.5	≤0.03
**11689**	dog	NA	NA	NA	ear	Siófok	≤0.03	0.06	16	0.5	≤0.03
**11715**	cat	European shorthair	male	5 months	ear	Budapest	0.03	1	16	1	0.25
**11721**	dog	Chihuahua	female	11 years	ear	Budapest	≤0.03	0.125	8	1	0.125
**11768**	dog	German Shepherd	female	5 years	ear	Komárom	0.5	0.25	16	2	≤0.03
**11819**	dog	Pug	female	11 years	ear	Budapest	≤0.03	0.03	8	1	0.25
**11820**	dog	West Highland White Terrier	female	11 years	ear	Martonvásár	0.125	1	16	1	0.25
**11823**	dog	West Highland White Terrier	male	3 years	ear	Érd	0.125	1	16	1	0.25
**11870**	dog	Yorkshire Terrier	female	1 years	ear	Budapest	0.06	0.125	8	1	0.5
**11872**	dog	NA	NA	NA	ear	Budapest	0.06	1	8	1	0.5

**11900**	dog	West Highland White Terrier	female	5 years	ear	Paks	0.06	0.5	16	0.5	0.03
**11927**	dog	NA	NA	NA	ear	Budapest	0.06	0.25	8	1	0.06
**11938**	dog	Toy Poodle	female	13 years	ear	Miskolc	0.06	1	4	8	0.125
**11960**	dog	NA	female	3 years	ear	Kóny	0.06	0.125	2	1	0.06
**11995**	dog	NA	NA	NA	ear	Felsőpakony	0.06	0.03	2	1	0.06
**12003**	dog	Bernese Mountain Dog	male	1 years	ear	Komárom	0.125	1	4	1	0.125
**12004**	dog	NA	NA	NA	ear	Hajdúszoboszló	0.06	0.06	8	1	0.125
**12067**	dog	English Cocker Spaniel	male	13 years	ear	Érd	0.06	0.25	4	1	0.06
**12069**	dog	Pomeranian	male	1 years	ear	Budapest	0.25	0.125	16	4	0.25
**12070**	dog	Hungarian Pointer	male	4 years	ear	Budapest	0.06	0.06	4	1	0.125
**12090**	dog	Yorkshire Terrier	female	13 years	ear	Budapest	0.06	0.03	4	0.5	0.06
**12188**	dog	NA	NA	NA	ear	Debrecen	≤0.03	0.06	2	0.5	0.5
**12220**	dog	Cross-breed	female	13 years	ear	Szentendre	0.06	≤0.03	4	0.5	0.25
**12232**	dog	Husky	male	4 years	ear	Dunakeszi	0.06	0.03	4	1	0.25
**12346**	dog	Chow Chow	male	3 years	ear	Érd	0.06	0.125	8	1	0.25
**12315**	dog	Hungarian Pointer	male	NA	ear	Budapest	0.125	0.125	16	2	0.25
**12249**	dog	Cross-breed	NA	NA	ear	Kapuvár	0.06	0.125	8	2	0.125
**12352**	cat	Maine Coon	female	5 years	ear	Budapest	0.06	0.5	4	1	0.25
**12372**	dog	Bulldog	male	13 years	ear	Budapest	0.06	0.06	4	0.5	0.125
**12373**	dog	German Boxer	male	3 years	ear	Miskolc	0.125	0.5	32	2	0.06
**12405**	dog	Bichon Havanese	female	8 years	ear	Martonvásár	0.125	0.06	8	1	0.25
**12408**	cat	European shorthair	male	11 years	ear	Veszprém	0.03	0.125	2	0.5	≤0.03
**12416**	dog	Puli	male	NA	ear	Budapest	0.125	0.25	8	0.5	≤0.03
**12438**	dog	NA	NA	NA	ear	Hajdúszoboszló	0.06	0.5	4	1	0.25
**12581**	dog	Labrador Retriever	male	4 years	ear	Mór	0.06	0.5	8	1	0.25
**12482**	dog	Bichon	NA	3 months	ear	Budapest	0.125	0.125	8	2	0.25
**12528**	dog	Poodle	female	7 years	ear	Nyíregyháza	0.03	0.5	4	0.5	0.06
**12529**	dog	Poodle	female	NA	ear	Eger	0.06	0.06	8	1	0.06
**12735**	dog	NA	NA	NA	ear	Dunakeszi	0.06	0.125	4	0.5	0.03
**12736**	dog	Boston Terrier	female	6 years	ear	Budapest	0.06	0.25	4	1	0.5
**12745**	dog	NA	NA	NA	ear	Budapest	≤0.03	0.25	4	1	0.5
**12597**	dog	Bernese Mountain Dog	female	2.5 years	ear	Győr	≤0.03	0.25	4	1	1

**12730**	dog	NA	NA	NA	ear	Dunakeszi	≤0.03	0.125	4	1	0.25
**12695**	dog	Briard	female	8 years	ear	Budapest	0.06	0.5	32	2	0.03
**12693**	dog	Bernese Mountain Dog	female	5 years	ear	Siófok	0.25	0.5	>32	16	0.125
**12639**	dog	NA	NA	NA	ear	Debrecen	≤0.03	0.06	8	0.5	0.06
**12598**	cat	NA	male	7 years	ear	Budapest	0.06	0.5	8	0.5	0.125
**12747**	dog	Bulldog	male	NA	ear	Budapest	≤0.03	0.03	4	1	0.25
**12799**	dog	NA	female	13 years	ear	Budapest	0.06	0.25	8	1	1
**12861**	dog	Yorkshire Terrier	female	12 years	ear	Nyíregyháza	0.06	1	32	0.5	0.06
**12985**	dog	Cross-breed	male	3 years	ear	Pécs	0.06	0.5	4	1	0.125
**13029**	dog	Labrador Retriever	female	9 years	ear	Budapest	0.25	0.25	4	1	≤0.03
**13119**	dog	NA	NA	6 years	ear	Budapest	0.06	0.5	16	1	0.5
**13144**	dog	Dachshund	male	5 years	ear	Veresegyház	0.125	0.5	8	1	0.25
**13171**	dog	Boxer	male	5 years	ear	Sopron	0.125	1	8	2	0.25
**13172**	dog	English Cocker Spaniel	male	4 years	ear	Paks	0.06	0.5	16	2	0.25
**13025**	dog	Bichon Havanese	male	6 years	skin	Budapest	0.06	0.25	4	1	0.125
**13070**	dog	Bernese Mountain Dog	male	3 years	ear	Eger	0.03	0.5	8	1	≤0.03
**13313**	dog	NA	NA	2 years	ear	Budapest	0.125	0.25	8	2	0.25
**13327**	dog	Labrador Retriever	male	1 years	ear	Csömör	0.125	1	8	2	0.25
**13239**	dog	Miniature Poodle	female	11 years	ear	Budapest	0.06	0.25	8	1	≤0.03
**13375**	dog	Cross-breed	male	4 years	ear	Érd	0.06	0.5	4	0.5	0.25
**13383**	dog	Dogue de Bordeaux	male	2 years	ear	Kecskemét	0.06	1	2	1	0.06
**13400**	dog	Poodle	female	2 years	ear	Budapest	0.125	0.25	8	2	0.25
**13381**	dog	Cocker Spaniel	male	9 years	ear	Budapest	0.06	0.125	4	1	0.06
**13331**	cat	NA	NA	8 years	ear	Siófok	≤0.03	0.125	16	0.5	0.06
**13398**	dog	NA	NA	NA	ear	Budapest	0.125	0.25	8	2	0.125
**13428**	dog	Boston Terrier	NA	NA	ear	Tatabánya	0.25	0.5	16	4	0.125
**13189**	dog	Komondor	male	4 years	ear	Herceghalom	≤0.03	0.125	16	0.5	≤0.03

CLT: clotrimazole. ITR: itraconazole. KTZ: ketoconazole. MCZ: miconazole. NA: not available. TER: terbinafine
